# Text4PTSI: A Promising Supportive Text Messaging Program to Mitigate Psychological Symptoms in Public Safety Personnel

**DOI:** 10.3390/ijerph20054215

**Published:** 2023-02-27

**Authors:** Gloria Obuobi-Donkor, Reham Shalaby, Ejemai Eboreime, Belinda Agyapong, Natalie Phung, Scarlett Eyben, Kristopher Wells, Carla Hilario, Raquel da Luz Dias, Chelsea Jones, Suzette Brémault-Phillips, Yanbo Zhang, Andrew J. Greenshaw, Vincent Israel Opoku Agyapong

**Affiliations:** 1 Department of Psychiatry, Dalhousie University, Halifax, NS B3H 4R2, Canada; 2 Department of Psychiatry, University of Alberta, Edmonton, AB T6G 2R3, Canada; 3 Operational Stress Injury Clinic, Alberta Health Services, Edmonton, AB T5J 3E4, Canada; 4 Department of Child and Youth Care, Faculty of Health and Community Studies, MacEwan University, Edmonton, AB T5J 4S2, Canada; 5 School of Nursing, Faculty of Health and Social Development, The University of British Columbia, Kelowna, BC V1V 1V7, Canada; 6 Department of Occupational Therapy, Faculty of Rehabilitation Medicine, University of Alberta, Edmonton, AB T6G 2R3, Canada

**Keywords:** Text4PTSI, text messaging, mental health, public safety personnel

## Abstract

Background: Public safety personnel experience various mental health conditions due to their work’s complex and demanding nature. There are barriers to seeking support and treatment; hence, providing innovative and cost-effective interventions can help improve mental health symptoms in public safety personnel. Objective: The study aimed to evaluate the impact of Text4PTSI on depression, anxiety, trauma, and stress-related symptoms, and the resilience of public safety personnel after six months of providing supportive text message intervention. Methods: Public safety personnel subscribed to Text4PTSI and received daily supportive and psychoeducational SMS text messages for six months. Participants were invited to complete standardized self-rated web-based questionnaires to assess depression, anxiety, posttraumatic stress disorder (PTSD), and resilience symptoms measured on the Patient Health Questionnaire-9 (PHQ-9), Generalized Anxiety Disorder-7 scale (GAD-7), Posttraumatic Stress Disorder Checklist-Civilian Version (PCL-C), and the Brief Resilience Scale (BRS), respectively. The assessment of mental health conditions was conducted at baseline (enrolment) and six weeks, three months, and six months after enrollment. Results: One hundred and thirty-one subscribers participated in the Text4PTSI program, and eighteen completed both the baseline and any follow-up survey. A total of 31 participants completed the baseline survey and 107 total surveys were recorded at all follow-up time points. The baseline prevalence of psychological problems among public safety personnel were as follows: likely major depressive disorder (MDD) was 47.1%, likely generalized anxiety disorder (GAD) was 37.5%, low resilience was 22.2%, and likely PTSD was 13.3%. At six months post-intervention, the prevalence of likely MDD, likely GAD, and likely PTSD among respondents reduced; however, a statistically significant reduction was reported only for likely MDD (−35.3%, X^2^ (1) = 2.55, *p* = 0.03). There was no significant change in the prevalence of low resilience between baseline and post-intervention. There was a decrease in the mean scores on the PHQ-9, GAD-7, PCL-C, and the BRS from baseline to post-intervention by 25.8%, 24.7%, 9.5%, and 0.3%, respectively. However, the decrease was only statistically significant for the mean change in GAD-7 scores with a low effect size (t (15) = 2.73, *p* = 0.02). Conclusions: The results of this study suggest a significant reduction in the prevalence of likely MDD as well as the severity of anxiety symptoms from baseline to post-intervention for subscribers of the Text4PTSI program. Text4PTSI is a cost-effective, convenient, and easily scalable program that can augment other services for managing the mental health burdens of public safety personnel.

## 1. Introduction

First responders comprise police, paramedics, firefighters, ambulance personnel, and emergency medical personnel who have acquired the skills and training and are the first contact to assist individuals during emergencies such as accidents and fire outbreaks [1,2]. Public safety personnel, on the other hand, ensure safety. Public safety personnel include but are not limited to first responders, military personnel, and law enforcement agents, among others [3,4]. All these personnel are included in the definition of public safety personnel and have been extensively studied [1,3,4]. Due to the scope of their work, they are exposed to distressing experiences and are predisposed to physical and mental health risk factors [5]. It is unsurprising that most of the literature consistently reports increased stress-related psychopathology among first responders [3,6,7]. Although mental health disorders among public safety personnel range from 10% to 35% internationally, these values vary based on specific occupations and the type of mental health condition [8].

Recent literature has studied public safety personnel, especially emergency health workers, military personnel, firefighters, and law enforcement. Published literature has recorded a prevalence of any mental health condition from 6.4% to 57% for firefighters [9,10], 3.72% to 37.8% for military personnel [10,11,12], and 5.8% to 19.6% for police officers [9,13,14]. Prevalence is dynamic and these range of prevalence could be attributed to sample size, population, methodology, or time [10]. Some reports may study one group of personnel while other studies research public safety personnel in general, which may cause the variation in the prevalence.

Researchers have also reported higher rates of psychological distress, such as posttraumatic stress, depression, anxiety, sleep disorders, and mild traumatic brain injury among these groups [10]. For example, among police officers, depression and anxiety rates are 14.6% and 9.6%, respectively [14]. The point prevalence of depression (16–26%) is higher than the general population, with a rate of 7% [4,15,16]. A systematic review and meta-analysis estimated the prevalence rates of depression and anxiety at 15% among ambulance personnel [17]. A study conducted among rescue workers demonstrated that trauma-exposed disaster personnel have a 16% prevalence of depression between 7 and 13 months post-disaster [18]. Similarly, 5–32.8% of firefighters reported depressive symptoms, and 3.8–30.5% reported anxiety symptoms in China [19].

Posttraumatic stress disorder (PTSD) is one of the most common mental health conditions experienced following traumatic events and is widely studied among public safety personnel [10,13,20]. The Diagnostic and Statistical Manual of Mental Disorders-5 (DSM-5) identifies that PTSD is higher among veterans and other public safety personnel such as the police, firefighters, and emergency medical personnel [21]. Moreover, individuals may be symptomatic of PTSD even if they do not meet all the diagnostic criteria [22]. A study in Canada reported that 7.6% of police officers experience full PTSD, while 6.8% experience partial PTSD after exposure to traumatic experiences at work [23]. Research literature estimates the prevalence of PTSD at 10% in public safety personnel, which is higher than the general population, estimated between 1.3% and 3.5% [24,25,26]. Low resilience to psychopathology is another challenge for most public safety personnel [27]. The ability to continue functioning after a traumatic event is usually a characteristic of successful adaptation and coping [27,28].

The increasing prevalence of mental health symptoms among public safety personnel is alarming. Additionally, the ongoing exposure to traumatic events throughout public safety personnel careers warrants interventions that can be easily accessed. Evidence suggests that public safety personnel are hesitant to access mental health services due to cost and stigma; hence, outreach programs are recommended [26]. E-mental health initiatives have proven promising in improving the utilization of mental health services in Canada [29]. Interventions that are cost-effective and helpful are highly recommended, given that most individuals with mental health conditions do not access services due to stigma or geographical barriers [30]. Cognitive behavioral therapy (CBT), which is a form of psychological treatment that helps to change the negative thought patterns of an individual [31], when delivered through text messaging has been proven to bridge the gap in mental health interventions and reduce anxiety and depression symptoms [32,33,34]. Text message systems, invented in the 1990s, are an affordable communication method that is a normal part of most people’s lives today and can be used to deliver innovative healthcare [35].

Most studies have reported a reduction in psychopathology when delivering supportive care through text message intervention. For example, a study of text messaging used as part of a clinical intervention reported a significant improvement in psychological symptoms compared to the usual care in ten out of sixteen randomized controlled trials [36]. Similarly, a systematic review and meta-analysis utilizing randomized control trials showed that text messaging reduces depression symptoms among people living with clinical depression [37]. Another study found that text messaging has the potential to reduce depression symptoms among adults with predominant depression [38]. Similar text messaging programs, Text4Hope and Text4Mood, achieved significant reductions in anxiety and depression symptoms among subscribers [32,39,40].

Supportive text message therapy has also been effective in mood disorders [40].

Text4PTSI is a supportive daily text messaging program based on CBT and trauma therapy principles and aims to provide mental health support for public safety personnel [2]. The program was initiated to prevent, manage, and reduce psychopathology in this cohort. This paper evaluates the impact of Text4PTSI on depression, anxiety, posttraumatic stress symptoms, and low resilience six months post-intervention.

We hypothesize that participants who enroll in the program will have improved depression, posttraumatic stress symptoms, and anxiety symptoms, and improved resilience in comparison to their baseline parameters.

## 2. Methodology

### 2.1. Study Design/Ethical Considerations

This longitudinal study assessed the effectiveness of a supportive text message intervention program (Text4PTSI) after participants received six months of daily supportive text messages. The Health Research Ethics Board (HREB) of the University of Alberta approved the study (Pro00108966). Informed consent was implied if respondents reviewed the associated information leaflet and completed and submitted the voluntary online surveys. Confidentiality and data security measures were adhered to as approved by the HREB.

### 2.2. Data Collection

Data were collected through online self-administered questionnaires with the REDCap software program [41]. The procedure for data collection is fully described in the study protocol [2]. The targeted population was public safety personnel, including emergency department health workers, paramedics, firefighters, and police/law enforcement agents. Participants subscribed to the program by texting “PTSI” to a designated phone number and were enrolled in the program. Participants received daily unidirectional supportive text messages for a period of six months. An example of a message is:

*Letting go of resentment is a gift you give yourself, and it will ease your journey immeasurably. Make peace with everyone, and happiness will be yours. Trauma can feel like a gloomy cloud over all areas of your life. The first step in treatment is to understand what trauma is, the symptoms, and how and why it is treated.*

Participation in the program was voluntary, and supportive text messages were received irrespective of survey completion. A maximum of ten minutes was required to complete the survey. Participants could opt out of the program anytime by texting the word “STOP” to the same number that sends the supportive messages. No incentives were offered for survey participation.

**Figure 1:**
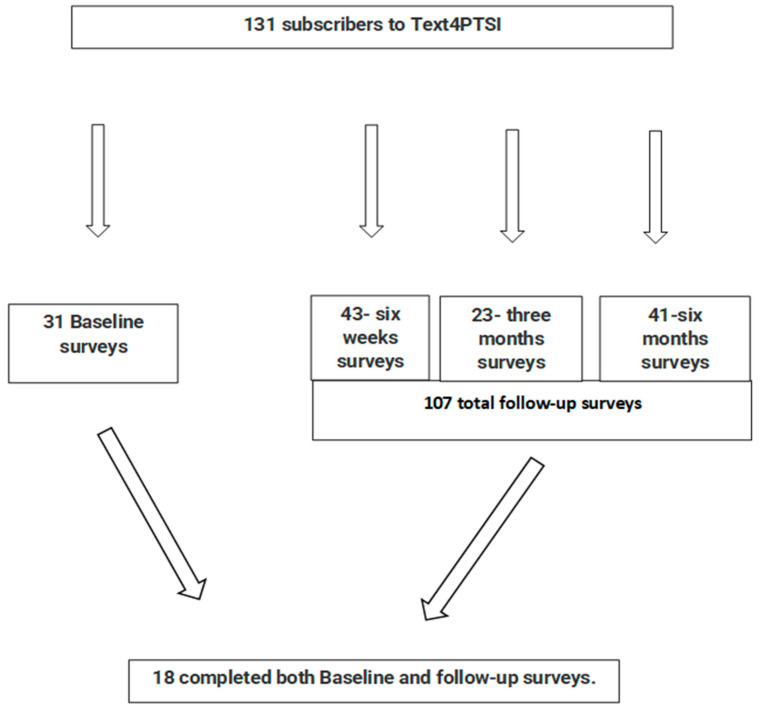
Study flow chart.

At enrollment, the baseline survey captured subscribers’ demographic and clinical information. Follow-up survey links were sent via text message to the participants’ mobile phones six weeks, three months, and six months after enrollment. Figure 1 illustrates the participants’ flowchart and the number of surveys collected at each time point.

### 2.3. Outcome Measures

The primary outcome measure was the mean difference in scores on the Patient Health Questionnaire-9 (PHQ-9) scale, the Generalized Anxiety Disorder-7 scale (GAD-7), the Brief Resilience Scale (BRS), and the Posttraumatic Stress Disorder Checklist-Civilian Version (PCL-C) at baseline and follow-up (six weeks, three months, and six months) and the changes in the prevalence of self-reported posttraumatic stress, depression, anxiety, and low resilience symptoms at the various follow-up time points from baseline. The prevalence was calculated by dividing the total number of participants who reported the likelihood of the clinical condition by the total eligible number of participants. The PHQ-9, GAD-7, BRS, and PCL-C scales assessed depression, anxiety, low resilience, and posttraumatic stress symptoms, respectively.

The PHQ-9 is a self-report instrument used to inform screening, diagnosing, monitoring, and measuring the severity of depression. It is a nine-item questionnaire measured on a four-point Likert scale from “0” (not at all) to “3” (nearly every day) [42]. The PHQ-9 scale categorizes depression scores into none to minimal (0–4 points), mild (5–9 points), moderate (10–14 points), moderately severe (15–19 points), and severe (20–27 points) [42]. The PHQ-9 reliability and validity have demonstrated good psychometric qualities. The PHQ-9 has a high internal consistency: Cronbach alphas of 0.86 and 0.89 were reported in two distinct studies [42]. For analysis purposes, they were recategorized into two variables: none to mild depression (a score ≤ 9) and moderate to severe depression (a score ≥ 10).

The GAD-7 is a self-report instrument used to inform screening, diagnosing, monitoring, and measuring the severity of anxiety symptoms. The scale is a 4-point Likert-type scale (0—not at all, 1—several days, 2—more than half the days, and 3—nearly every day). Scores range from 0 to 21. The scale categorizes depression scores as minimal (0–4), mild (5–9), moderate (10–14), and severe anxiety (15–21) [43]. The GAD-7 scale demonstrates consistency and a one-factor structure in its usage globally, and all its items represent one construct [44,45]. The tool has acceptable test–retest reliability and is a validated tool for assessing the severity of GAD symptoms in research and clinical practice [42,45]. The scores were recategorized into low anxiety (score < 10) and moderate to high anxiety (score ≥ 10).

The BRS assesses resilience as one’s ability to bounce back or recover from stressful moments [46]. The BRS is a 6-item questionnaire measured on a five-point Likert scale, and a total score can range from 6 to 30. An average score < 3 indicates low resilience, while an average score > 3 denotes normal to high resilience. The literature has demonstrated adequate internal consistency of the BRS, with Cronbach alphas ranging from 0.80 to 0.90 and a fair test–retest reliability coefficient [46].

The PCL-C was used to assess the likely PTSD symptoms of participants [47]. This scale is a 17-item checklist measured on a 5-point Likert scale; each symptom is rated from 1 (not at all) to 5 (extremely). A total score ranges from 17 to 85, with a higher score indicating a severe form of PTSD. A score ≥ 44 denotes likely PTSD, while ≤44 shows unlikely PTSD. The PCL-C demonstrates high internal consistency (Cronbach’s = 0.94) and acceptable reliability and convergent validity [48].

#### Sample Size Considerations

With a projection that the effect size for the reduction in mean PCL-C, GAD-7, and PHQ-9 scores at six months from baseline would be 0.1, a population variance of 1.0 for each scale mean score, a two-sided significance level α = 0.05, and a power of 90% (β = 0.1), using an online script [49], we estimated that the sample size needed to assess the effects of the daily supportive text messages on the outcome variables would be 48.

### 2.4. Statistical Analysis

Data were analyzed utilizing SPSS Version 26 [50] using descriptive and inferential statistics. Demographic characteristics were compared using chi-square or Fisher’s exact tests between two samples of study participants who responded to the baseline survey and at least one follow-up survey (sample A, n = 18) and participants who responded only to one survey, i.e., only the baseline, six weeks, three months, or six months survey (sample B, n = 69). This was to determine how similar or dissimilar the sociodemographic characteristics of the two samples were.

In order to evaluate the generalizability of the data, the baseline prevalence of the four mental health conditions under study was appraised among the participants who completed both the baseline survey and follow-up (n = 18) and participants who completed only the baseline surveys (n = 11) (two responses did not provide data related to the clinical conditions under study).

With the same concept, the follow-up prevalence was assessed between the responses of those who completed the baseline and follow-up surveys (n = 18) and the responses of those who responded only to the follow-up surveys (107).

Regarding the outcome assessment, the ongoing analysis was run on sample A (the participants who completed both baseline and follow-up surveys) to examine the outcome scales under study. The last observation carried forward (LOCF) method was applied, i.e., the latest response was recorded (six months). The last observation (six weeks or three months) was carried forward (imputed) when the latest response was missing [51]. In order to appraise the outcome measures, paired sample t-tests were used to assess differences between the mean PHQ-9, GAD-7 BRS, and PCL-C scores at baseline and follow-up for participants who completed the online scales at baseline and any follow-up time points. The chi-square test was employed to compare the prevalence of likely MDD, GAD, low resilience, and PTSD symptoms at baseline and follow-up.

Moderate to severe depression and GAD were assessed using a score of ≥10 on the PHQ-9 and GAD-7 scales. Low resilience and likely PTSD were assessed using the BRS score of <3 and PCL-C score of ≥44, respectively. The total number of responses was reported and represented for each variable.

## 3. Results

### 3.1. Demographic Characteristics

A total of 131 subscribers participated in Text4PTSI, providing 138 surveys over all the study time points, and 31 and 107 surveys were provided at baseline and total of the three follow-up surveys, respectively. Out of the 131 subscribers, 18 completed baseline and follow-up surveys.

Table 1 illustrates the distribution of demographic characteristics of all the study participants who responded to the baseline and at least one more survey and the participants who responded to only one survey. The bivariable analysis did not show a statistically significant association between the sociodemographic characteristic of sample A and sample B (*p* ≤ 0.05). This implies that the two samples were similar in terms of participants’ sociodemographic characteristics. The results presented in Table 1 also suggest that 37.2% of the sample were police/law enforcement agents, and most of the participants were between the ages of 31 and 60 (56/80, 70%). They were predominantly females (47/81, 58%), white (63/81, 77.7%), had attained postsecondary education (68/81, 83.9%), had employment (71/81, 87.7%), were in a relationship (58/81, 72.5%), and owned their homes (57/81, 70.4%).

In order to evaluate the generalizability of the data, based on likely MDD, GAD, low resilience, and likely PTSD at baseline (Table 2), we compared the clinical characteristics between participants who completed both the baseline survey and follow-up and participants who completed only the baseline surveys, as indicated in Table 2A. The data did not find any statistically significant difference between the groups (baseline and follow-up and only baseline) for likely MDD, GAD, and low resilience (*p* > 0.05). However, the likelihood of PTSD was significantly higher in participants who completed the baseline survey only compared to sample A. These results imply that at baseline, the likelihood of mental health conditions under study was similar between study participants and subscribers who did not complete the follow-up survey, except for likely PTSD. Likewise, participants who completed the baseline and follow-up surveys were examined against participants who completed only the follow-up survey (Table 2B). There was no statistically significant difference between the two groups for all outcome scales. These results suggest that after receiving the intervention, the mental health symptoms were, for the most part, similar for participants who completed only the follow-up and those who completed both the baseline and the follow-up.

### 3.2. Analysis of Study Outcomes

The ongoing analysis was run on sample A (the participants who completed both surveys, baseline and any follow-up survey). Table 3 describes the change in prevalence of the four mental health conditions under study after introducing Text4PTSI for six months. As illustrated in Table 3, likely MDD recorded a 35.3% significant decrease in prevalence from baseline to six months (*p* = 0.03). There were reductions in the prevalence of likely GAD and likely PTSD (18.7% and 6%, respectively), but these did not achieve statistical significance. Similarly, the prevalence of low resilience did not show any significant difference between the baseline and follow-up prevalence.

Table 4 shows the changes in the mean scores of the primary outcome measures after six months from baseline for participants who completed both the baseline and other follow-up surveys (sample A). As indicated in Table 4, the participants recorded lower mean scores on the PHQ-9, GAD-7, PCL-C, and BRS scales at follow-up compared to the mean scores at baseline, suggesting improvement in depression, anxiety, resilience, and PTSD symptoms. However, only the GAD-7 change in mean scores from baseline was statistically significant (t (15) = 2.73, *p* = 0.02), with a low effect size (Cohen’s d = 0.38).

## 4. Discussion

Public safety personnel may experience psychological distress due to their work’s challenging and complex nature. The Text4PTSI program is a structured cognitive behavioral therapy-based text messaging program designed to prevent and reduce the occurrence of MDD, GAD, PTSD, and low resilience among Canada’s public safety personnel. These cohorts received daily supportive text messages for six months. Our results showed a significant reduction in mean scores of only the GAD symptoms. However, there was an observed reduction noted in other mean scores, including for scales used to measure depression, resilience, and posttraumatic stress symptoms, even though they did not reach statistical significance. Additionally, the results showed a reduction in the prevalence of mental health conditions from the initial baseline survey results. Again, it was only the change in prevalence of likely MDD which was statistically significant, representing a 35.3% reduction from the established baseline. The changes in prevalence of likely GAD and PTSD were 18.7% and 6.6%, respectively, albeit not significant, and there was no significant reduction in the prevalence of low resilience at the six-month follow-up for participants. The results of this study also indicate that there was no substantial difference in the prevalence of the mental health burden of participants who completed baseline and follow-up and those who completed follow-up or baseline only, suggesting the potential generalizability of the results over the whole study sample.

The baseline prevalence of self-reported depression, anxiety, and posttraumatic stress symptoms in our study was recorded at 47.1%, 37.5%, and 13.3%, respectively, which is higher than what has been reported in other studies among public safety personnel [1,18,52]. A recent meta-analysis among first responders reported a pooled prevalence of depression of 31% [1] and 15% among ambulance personnel [17]. Similarly, a case–control analysis of emergency medical technicians recorded a 6.8% prevalence of depression [53]. Likewise, the prevalence of PTSD ranged from 3.7% to 12.6% among military personnel [12,54]. Anxiety symptoms recorded in other studies have also been lower than reported in this study [55,56]. The prevalence of anxiety among firefighters, EMTs, and paramedics ranged from 4.2% to 26% [55,56]. The variation in prevalence may be attributed to the type of public safety personnel group, scales used, and location [8,57]. While other studies focus on a particular public safety personnel group, our study focused on different personnel, hence the variation in prevalence. A study in Canada among public safety personnel reported elevated symptoms of various mental disorders despite prior treatments, and the public safety personnel experienced reductions in their symptoms following internet-delivered cognitive behavioral therapy (iCBT) [58].

After six months of receiving the supportive text message intervention, the prevalence of mental health burden was reduced in subscribers of Text4PTSI. Although not all subscribers may have read the supportive text messages they received, it is possible that most people who read the text benefited from the program. The most significant reduction was for MDD symptoms, with a prevalence reduction of 35.3%, and a 25% reduction in mean scores on the GAD-7 scale from baseline. This study’s findings are consistent with previous research, which demonstrated that novel interventions, including supportive text messaging and internet-based CBT approaches, can be effective and have greater efficacy in preventing and managing mental health conditions among public safety personnel [59,60,61]. Although the effect sizes (Cohen’s d) recorded in this study were small, the literature reveals that interventions without a therapist directly involved record a smaller effect size than an intervention with a therapist involved [39,62]. Since there was no control group, the improvement and related effect sizes could also be attributed to other factors such as the possibility of spontaneous remission over the 6-month period.

The findings from this study indicate that the prevalence of anxiety and depression symptoms significantly reduced after receiving the intervention. The reduction in anxiety symptoms recorded an effect size of 0.38, with a change in the mean score of −2.07. This finding agrees with outcomes reported from the Text4Hope program, which delivered supportive text messages to individuals during the COVID-19 pandemic and showed that anxiety symptoms reduced after the intervention [32]. A meta-analysis of randomized controlled trials reported an effect size of 0.35 for anxiety symptom reduction for a sample that received iCBT [63]. Furthermore, a systematic review to assess the efficacy of text message interventions reported that the intervention improves anxiety symptoms with a fair effect size [64].

Although there was no statistically significant improvement in depression symptom severity as measured by the PHQ-9 scale from baseline to post-intervention in this study, there was a significant reduction in the prevalence of likely MDD post-intervention. The later finding is consistent with other psychological interventions targeting the general population and public safety personnel [65,66], and several studies involving the delivery of support via text messages or internet-delivered CBT have recorded improved depression symptoms after the intervention [39,67,68,69]. For example, a study among Canadian public safety personnel reported that participants experienced decreased depression symptoms, anxiety, and posttraumatic stress symptoms after introducing an iCBT program tailored for public safety personnel [69].

This study did not find a significant change in the prevalence of low resilience from baseline to post-intervention. Resilience has been described as the ability to “bounce back” from adversity impacting mental health [70]. In contrast, improved resilience may improve health symptoms [71]. Hence, participants may have developed resilience to mental health symptoms, which may explain why there was no statistically significant change in the BRS scores. Moreover, the study cohort showed mostly normal to high resilience before the intervention (BRS > 3), which did not deteriorate after the intervention. Perhaps the Text4PTSI program was not effective enough in addressing low resilience symptoms in the 22% of the study population.

Our study also did not record a significant change in either the severity or prevalence of likely PTSD symptoms in respondents from baseline to post-intervention. It is possible that the duration of the intervention could have resulted in this non-significant change in comparison to iCBT treatments delivered for a more extended period which have been reported to be effective in reducing PTSD symptoms [72]. However, our findings showed a statistically significant difference in the prevalence of PTSD, where the participants who completed both baseline and follow-up surveys were less likely to report PTSD symptoms compared to those who completed only the baseline survey. There is a possibility that participants who were experiencing more PTSD symptoms were not encouraged enough to respond to the follow-up survey, irrespective of the intervention they received. They probably found intervention either unhelpful or that it exacerbated their symptoms.

Moreover, 70% of our participants were between 31 and 60 years old, which may have affected the utilization of the service. Individuals born after the 1980s (often referred to as digital natives) may have more exposure to digital technologies than older people, which may be a barrier to completing online surveys and navigating digital media and other digital technologies [73,74,75].

## 5. Limitations

There are limitations to this study which need to be considered when interpreting the findings. First, although validated and standardized scales were used in this study, the self-reported questionnaires are nonetheless non-diagnostic. Second, the relatively small sample size of participants who completed both the baseline and follow-up surveys (18/131) makes it likely that this study was underpowered to detect significant differences from baseline to post-intervention. In addition, the low response rate, which is potentially a result of the online nature of the surveys, could impact the generalizability of our findings to all subscribers of Text4PTSI. Surveys delivered via text messages are nearly 15% less likely to retain participants for follow-up assessment compared to other mediums such as paper surveys [76,77]. However, the comparative analysis for the total cohort at baseline and follow-up revealed mostly non-significant differences, suggesting the potential generalizability of the results to all subscribers. Considering all the follow-up surveys (6 weeks, three months, and six months) together instead of analyzing them differently may have led to a loss of precision in the results and the interpretations. Additionally, the absence of a control group who did not receive the intervention led to uncertainty in concluding whether the effects found are due to the intervention or other factors such as time progression (regression to the mean).

Again, the effect sizes for significant results in this study were small, which may minimize the strength of our findings. Nevertheless, the literature records small effect sizes for self-help interventions that do not directly involve a therapist [39,61,68]. Future studies adopting text message intervention can encourage public safety personnel to subscribe and complete follow-up assessments through an incentive package since the literature reports that even small monetary value incentives promote response rates in research [78].

## 6. Conclusions

The current Text4PTSI study shows a substantially higher prevalence of depression, anxiety, posttraumatic stress symptoms, and low resilience among public safety personnel compared with the general public. The results from our program showed a reduction in the mean score for the GAD scale and a statistically significant reduction in the prevalence only of MDD at six months from baseline. The statistically significant improvements in depression symptoms six months after the program suggest that there is a potential utility for Text4PTSI to be adopted as an innovative and cost-effective intervention for public safety personnel. Most public safety personnel are hesitant to access mental health services, and some have experienced barriers to adequately meeting their mental health needs [26]. The former may be due to cost, stigma, geographical constraints, or long wait times [29,30]. Considering these factors, proven technology-enabled CBT-based services such as Text4PTSI can address public safety personnel’s psychological and wellness needs. Future randomized control studies are recommended to comprehensively assess the impact of Text4PTSI on anxiety, depression, resilience, and PTSD symptoms in public safety personnel. It is also recommended that demographic differences be analyzed in future studies based on sex, gender identity, sexual orientation, race/ethnicity, and other similar factors. This may help target tailored text message interventions particularly to marginalized and vulnerable respondents.

## Figures and Tables

**Table 1:** Distribution of demographic characteristics of all the study participants among the participants who responded to baseline and at least one more survey (sample A) and the participants who responded to only one survey (sample B).

Variable	Sample A N = 18 (%)	Sample B N = 69 (%)	Total ^a^ N (%)	Chi-Squared /Fisher’s Exact ^b^	*p* Value
Public Safety Personnel					
Emergency department health workers	4 (22.2)	4 (6.7)	8 (10.3)	6.97 ^b^	0.11
Paramedics	4 (22.2)	11 (18.3)	15 (19.2)		
Firefighters	0 (0.0)	2 (3.3)	2 (2.6)		
Police/Law enforcement agents	8 (44.4)	21 (35.0)	29 (37.2)		
Other	2 (11.1)	22 (36.7)	24 (30.8)		
Age					
≤30 y	5 (29.4)	9 (14.3)	14 (17.5)	2.60	0.46
31–45 y	6 (35.3)	25 (39.7)	31 (38.8)		
46–60 y	5 (29.4)	20 (31.7)	25 (31.3)		
>60 y	1 (5.9)	9 (14.3)	10 (12.5)		
Gender					
Female	12 (66.7)	35 (55.6)	47 (58.0)	0.80 ^b^	0.72
Male	6 (33.3)	25 (39.7)	31 (38.3)		
Other	0 (0.0)	3 (4.8)	3 (3.7)		
Ethnicity					
White	12 (70.6)	51 (79.7)	63 (77.8)	7.79 ^b^	0.07
Indigenous	0 (0.0)	4 (6.3)	4 (4.9)		
Asian	1 (5.9)	7 (10.9)	8 (9.9)		
African descent	1 (5.9)	1 (1.6)	2 (2.5)		
Other	3 (17.6)	1 (1.6)	4 (4.9)		
Education Level					
High school diploma	2 (11.8)	10 (15.6)	12 (14.8)	0.57 ^b^	0.99
Postsecondary education	15 (88.2)	53 (82.8)	68 (84.0)		
Other	0 (0.0)	1 (1.6)	1 (1.2)		
Employment Status					
Employed	15 (88.2)	56 (87.5)	71 (87.7)	0.01	0.94
Unemployed	2 (11.8)	8 (12.5)	10 (12.3)		
Relationship Status					
In a relationship	11 (64.7)	47 (74.6)	58 (72.5)	6.32 ^b^	0.08
Single	3 (17.6)	14 (22.2)	17 (21.3)		
Separated or divorced	1 (5.9)	2 (3.2)	3 (3.8)		
Widowed	2 (11.8)	0 (0.0)	2 (2.5)		
Housing Status					
Own a home	12 (70.6)	45 (70.3)	57 (70.4)	0.58	0.82
Renting	3 (17.6)	14 (21.9)	17 (21.0)		
Living with family	2 (11.8)	5 (7.8)	7 (8.6)		

^a^ Total number represents the completed responses. ^b^ Fisher’s exact test.

**Table 2:** Distribution of prevalence of mental health conditions.

**A.** Distribution of baseline prevalence of mental health conditions between participants who completed only the baseline survey and participants who completed the baseline and any follow-up survey (sample A)					
**Variables, n (%)**	**Baseline and Follow-Up** **N (%)**	**Baseline Only** **N (%)**	**Total** **N (%)**	**Chi-Square**	***p*-Value**
Likely MDD ^a^	N = 17	N = 11	N = 28	0.15	0.70
At most mild depression	9 (52.9)	5 (45.5)	14 (50.0)		
Moderate to severe depression	8 (47.1)	6 (54.5)	14 (50.0)		
Likely GAD ^b^	N = 16	N = 11	N = 27	0.17	0.68
At most mild GAD	10 (62.5)	6 (54.5)	16 (59.3)		
Moderate to severe GAD	6 (37.5)	5 (45.5)	11 (40.7)		
Low resilience ^c^	N = 18	N = 11	N = 29	3.16	0.08
Normal to high resilience	14 (77.8)	5 (45.5)	19 (65.5)		
Low resilience	4 (22.2)	6 (54.5)	10 (34.5)		
Likely PTSD ^d^	N = 15	N = 10	N = 25	*	0.03
Unlikely PTSD	13 (86.7)	4 (40.0)	17 (68.0)		
Likely PTSD	2 (13.3)	6 (60.0)	8 (32.0)		
**B.** Distribution of follow-up prevalence of mental health conditions between participants who completed only follow-up and participants who completed the baseline and any follow-up survey (sample A)					
Variables, n (%) **	**Baseline and Follow-Up, N = 18 (%)**	**Follow-Up Only, ** **N = 56 (%)**	**Total** **N = 74 (%)**	**Chi-Square**	***p*-Value**
Likely MDD ^a^	N = 17	N = 54	N = 71	2.97	0.09
At most mild depression	15 (88.2)	36 (66.7)	51 (71.8)		
Moderate to severe depression	2 (11.8)	18 (33.3)	20 (28.2)		
Likely GAD ^b^	N = 16	N = 53	N = 69	0.58	0.45
At most mild GAD	13 (81.3)	38 (71.7)	51 (73.9)		
Moderate to severe GAD	3 (18.8)	15 (28.3)	18 (26.1)		
Low resilience ^c^	N = 18	N = 56	N = 74	1.42	0.23
Normal to high resilience	14 (77.8)	35 (62.5)	49 (66.2)		
Low resilience	4 (22.2)	21 (37.5)	25 (33.8)		
Likely PTSD ^d^	N = 16	N = 51	N = 67	0.39	0.53
Unlikely PTSD	12 (75.0)	34 (66.7)	46 (68.7)		
Likely PTSD	4 (25.0)	17 (33.3)	21 (31.3)		

* Fisher’s exact test was applied. ** Total numbers represent competed responses. ^a^ A cutoff score of ≥10 on the Patient Health Questionnaire-9 scale. ^b^ A cutoff score of ≥10 on the Generalized Anxiety Disorder-7 scale. ^c^ An average score < 3 on the Brief Resilience Scale. ^d^ A cutoff score of ≥44 on the PTSD Checklist-Civilian Version.

**Table 3:** Change in prevalence of likely GAD, and likely MDD, low resilience, and likely PTSD from baseline to follow-up.

Clinical Condition		Prevalence n (%)		Change from Baseline, %	X^2^ (df)	*p* Value
	N	Baseline	Follow-Up			
Likely MDD ^a^	17	8 (47.1)	2 (11.8)	−35.3	2.55 (1)	0.03
Likely GAD ^b^	16	6 (37.5)	3 (18.8)	−18.7	6.15 (1)	0.25
Low resilience ^c^	18	4 (22.2)	4 (22.2)	0	18.0 (1)	0.99
Likely PTSD ^d^	15	2 (13.3)	1 (6.7)	−6.6	1.29 (1)	0.99

^a^ A cutoff score of ≥10 on the Patient Health Questionnaire-9 scale. ^b^ A cutoff score of ≥10 on the Generalized Anxiety Disorder-7 scale. ^c^ An average score < 3 on the Brief Resilience Scale. ^d^ A cutoff score of ≥44 on the PTSD Checklist-Civilian Version.

**Table 4:** Changes in the mean scores of PHQ-9 and GAD-7 after the introduction of Text4PTSI.

Measure	Scores			Change from Baseline, %	Mean Difference (95% CI)	*p* Value	t Value (df)	Effect Size (Cohen’s d)
	n	Baseline Score, Mean (SD)	Follow-Up, Mean (SD)					
PHQ-9 ^a^	17	8.41 (5.46)	6.24 (4.98)	25.8	−2.17 (−0.45–4.81)	0.098	1.76 (16)	0.42
GAD-7 ^b^	16	8.38 (5.83)	6.31 (5.11)	24.7	−2.07 (−0.45–3.67)	0.02	2.73 (15)	0.38
BRS ^c^	18	3.29 (0.85)	3.28 (0.72)	0.3	−0.01 (−0.25–0.27)	0.94	0.08 (17)	0.01
PCL-C ^d^	15	32.13 (11.69)	29.07 (10.56)	9.5	−3.07 (−2.76–8.89)	0.28	1.13 (14)	0.28

^a^ PHQ-9: Patient Health Questionnaire-9. ^b^ GAD-7: Generalized Anxiety Disorder-7. ^c^ BRS: Brief Resilience Scale; ^d^ PCL-C: PTSD Checklist-Civilian Version.

## Data Availability

Data associated with this study will be made freely available upon reasonable request to the corresponding author.
